# Differential transmission of Sri Lankan cassava mosaic virus by three cryptic species of the whitefly *Bemisia tabaci* complex

**DOI:** 10.1016/j.virol.2019.11.013

**Published:** 2020-01-15

**Authors:** Yao Chi, Li-Long Pan, Sophie Bouvaine, Yun-Yun Fan, Yin-Quan Liu, Shu-Sheng Liu, Susan Seal, Xiao-Wei Wang

**Affiliations:** a Ministry of Agriculture Key Laboratory of Molecular Biology of Crop Pathogens and Insects, Institute of Insect Sciences, Zhejiang University, Hangzhou, 310058, China; b Natural Resources Institute, University of Greenwich, Chatham, Kent, ME4 4TB, UK

**Keywords:** Cassava mosaic disease, Sri Lankan cassava mosaic virus, *Bemisia tabaci*, Virus transmission, Differential transmission

## Abstract

In recent years, Sri Lankan cassava mosaic virus (SLCMV), a begomovirus (genus *Begmovirus*, family *Geminiviridae*) causing cassava mosaic disease in Asia, poses serious threats to cassava cultivation in Asia. However, the transmission of SLCMV in the areas into which it has recently been introduced remain largely unexplored. Here we have compared the transmission efficiencies of SLCMV by three widely distributed whitefly species in Asia, and found that only Asia II 1 whiteflies were able to transmit this virus efficiently. The transmission efficiencies of SLCMV by different whitefly species were found to correlate positively with quantity of virus in whitefly whole body. Further, the viral transmission efficiency was found to be associated with varied ability of virus movement within different species of whiteflies. These findings provide detailed information regarding whitefly transmission of SLCMV, which will help to understand the spread of SLCMV in the field, and facilitate the prediction of virus epidemics.

## 1 Introduction

Cassava (*Manihot esculenta* Crantz), normally grown for its starchy roots, is a staple food for nearly one billion people in 105 countries (http://www.fao.org/newsroom/en/news/2008/1000899/index.html as accessed on 10 April 2019). Thanks to its inherent tolerance to abiotic stresses such as drought and infertile soils, cassava is now being widely grown in tropical Africa, Asia and Latin America, making it one of the most important crops in the world (El-Sharkawy et al., 2004; Jarvis et al., 2012). More importantly, in the era of global warming, which is one of the major features of anthropogenic climate change in the near future, cassava is likely to be of increasing importance as a staple food (Jarvis et al., 2012). In recent decades, however, cassava mosaic diseases (CMDs) caused by cassava mosaic begomoviruses (CMBs), have emerged as a serious threat to the production of cassava. While significant yield losses have been documented due to CMD outbreaks, spread continues as evidenced by recent CMD emergence in Cambodia, Vietnam and China (Navas-Castillo et al., 2011; Rey et al., 2017; Uke et al., 2018; Wang et al., 2016, 2019). In light of the immediate threat caused by CMDs, research efforts are badly needed to identify the vector species and help to sustain the production of cassava in those affected and often the least developed regions.

So far, 11 CMBs have been shown to be the causal agents of CMDs, among which nine were found in Africa and two, namely Indian cassava mosaic virus (ICMV) and Sri Lankan cassava mosaic virus (SLCMV) were characterized in Asia (Legg et al., 2015). As for Asian CMBs, while ICMV was characterized earlier than SLCMV, SLCMV seemed to exhibit a wider geographical distribution and higher infectivity (Jose et al., 2011; Patil et al., 2004; Saunders et al., 2002). In the last few years, the threat of SLCMV has been evidenced by its rapid invasion of Cambodia, Vietnam and China (Uke et al., 2018; Wang et al., 2016, 2019). However, the transmission efficiency of SLCMV by different whitefly species remains hitherto unexplored.

Due to the fact that cassava plants are normally vegetatively propagated, inter-regional spread of CMBs entails the transport of infected cuttings (Legg et al., 2014). For example, the recent presence of SLCMV in China was attributed to the import of cassava cuttings from Cambodia (Wang et al., 2019). However, as learned from CMD epidemics in Africa caused by different CMBs, while infected cuttings serve as the initial source of infection, whitefly vectors can contribute to the secondary spread of the virus (Legg et al., 2011, 2014). Indeed, field surveys conducted in India and Vietnam have both shown that cutting-borne infections constitute a large proportion of CMD incidences in the field, followed by less frequent whitefly-borne infections (Jose et al., 2011; Minato et al., 2019). More importantly, transmission by whitefly will render some control strategies such as roguing and phytosanitary measures less effective, as epidemics are able to establish from a limited source of infection with the aid of whitefly vectors. Therefore, sustainable control of CMBs, including SLCMV, can only be achieved when a detailed understanding of whitefly transmission of CMBs, as well as alternative hosts is gained.

Begomoviruses are known to be vectored by the whitefly *Bemisia tabaci*, a species complex consisting of more than 36 genetically distinct but morphologically indistinguishable cryptic species (De Barro et al., 2011; Liu et al., 2012). For a given begomovirus, varied transmission efficiencies have been reported for different whitefly species, indicating different whitefly species may play varying roles in the epidemiology of certain begomoviruses (Beford et al., 1994; Li et al., 2010; Polston et al., 2014; Guo et al., 2015; Pan et al., 2018, Pan et al., 2018b; Wei et al., 2014; Fiallo-Olivé et al., 2019). Therefore, a detailed exploration on the transmission of begomoviruses by different whitefly species will lead to an improved understanding of the identity of vector species of the corresponding plant viral diseases, which will in turn facilitate the prediction of virus epidemics. This is exemplified by the case of cotton leaf curl Multan virus (CLCuMuV), wherein it was established that disease associated with this virus is primarily spread by Asia II 1, an indigenous whitefly species (Masood et al., 2018; Pan et al., 2018b).

In the present study, we characterized the transmission of SLCMV by three whitefly species of the *B. tabaci* complex found in the Asian SLCMV-affected regions (Götz and Winter 2016; Wang et al., 2016, 2019), namely Asia II 1, Mediterranean (MED) and Middle East-Asia Minor (MEAM1), and examined the factors involved. Firstly, we compared the transmission efficiencies of SLCMV by the three whiteflies species. Next, quantification of virus in whitefly whole body and honeydew was performed. Further, virus movement within whitefly body after virus acquisition was examined. These findings provide the first detailed whitefly transmission profile of a cassava mosaic begomovirus in Asia, based on which further implications are discussed.

## 2 Materials and methods

### 2.1 Plants and insects

In the present study, three kinds of plants, namely cotton (*Gossypium hirsutum* L. cv. Zhemian 1793), tobacco (*Nicotiana tabacum* L. cv. NC89) and cassava (*Manihot esculenta* cv. HLS11 and SC8) were used. All cotton and tobacco plants were grown in a greenhouses under natural lighting supplemented with artificial lighting at controlled temperatures of 25 ± 3 °C, 14 L: 10 D. For insects, three whitefly cryptic species, of which two are invasive worldwide including MED and MEAM1, one is indigenous species in Asia, namely Asia II 1, were used. These three whitefly species were chosen as they exhibit abundant distribution in regions where SLCMV occurred, including Vietnam, Cambodia and South China (Götz and Winter 2016; Uke et al., 2018; Wang et al., 2016, 2019; Hu et al., 2011) or have great potential to invade these regions (De Barro et al., 2011). All three whitefly species were originally collected from field in China between 2009 and 2012, and were maintained thereafter in the laboratory. The mitochondrial cytochrome oxidase I (mt*COI*) GenBank accession codes are GQ371165 (MED), KM821540 (MEAM1) and DQ309077 (Asia II 1). Whiteflies of all three species were maintained on cotton plants in separate insect-proof cages in artificial climate chambers at 26 ± 1 °C, 14 h light/10 h darkness and 60–80% relative humidity. The purity of each whitefly culture was monitored every three generations using the mt*COI* PCR-RFLP technique and sequencing as described before (Qin et al., 2013). In all experiments described in the present study, only female whiteflies with an age of 0–7 days post emergence were used.

### 2.2 Construction of infectious clones and agro-inoculation

SLCMV DNA A and DNA B were amplified from cassava samples collected from Cambodia (Wang et al., 2016) and were used to construct the infectious clones. The sequences of DNA A and DNA B of the isolate used for the construction of infectious clones have 3 point mutations compared to the original sequences (GenBank accession codes: KT861468 for DNA-A and KT861469 for DNA-B). We have presented the DNA sequence of SLCMV DNA A and DNA B in supplementary information. For DNA-A, full-length genome were amplified with primers SLCMV-A-FL-F and SLCMV-A-FL-R (HindIII restriction sites at both ends), and ligated into pGEM-T vectors (Promega, USA). Then 0.9 unit of DNA-A was amplified using the recombinant plasmids as template with SLCMV-A-0.9U-F (an AscI restriction site was introduced) and SLCMV-A-FL-R, and after digestion by HindIII and AscI, the fragments were inserted into the binary vector pBinPLUS to produce pBINPLUS-0.9A. Then the full-length genome of DNA-A was excised from T vectors by HindIII digestion and ligated into pBINPLUS-0.9A to produce pBinPLUS-1.9A. Similarly, the full-length genome of DNA-B was amplified with primers SLCMV-B-FL-F and SLCMV-B-FL-R (BamHI restriction sites at both ends), and ligated into pGEM-T vectors (Promega, USA). Then 0.9 unit of DNA-B was excised from the recombinant plasmids by digestion of BamHI and KpnI, and inserted into the binary vector pBINPLUS to produce pBINPLUS-0.9B. The full-length genome of DNA-B was excised from T vectors by BamHI digestion and ligated into pBinPLUS-0.9B to produce pBinPLUS-1.9B. The pBINPLUS-1.9A and pBINPLUS-1.9B plasmids were mobilized into the *Agrobacterium tumefaciens* strain EHA105 to obtain the infectious clones of SLCMV DNA-A and DNA-B. All primers were listed in Table 1.

**Table 1:** Primers used in this study.

Primer	Sequence (5′-3′)	Application
SLCMV-A-FL-F	CCCAAGCTTCGGAAGAACTCGAGTA	Amplification of full-length DNA-A
SLCMV-A-FL-R	CCCAAGCTTGAGTCTTCCGACAAAC	
SLCMV-A-0.9U-F	TTGGCGCGCCTTAGGGTATGTGAGGAATAT	Amplification of 0.9 unit of DNA-A
SLCMV-A-FL-R	CCCAAGCTTGAGTCTTCCGACAAAC	
SLCMV-B-FL-F	CGCGGATCCTATTAGACTTGGGCC	Amplification of full-length DNA-B
SLCMV-B-FL-R	CGCGGATCCAGATCCATGAGATATG	
SLCMV-PCR-F	CAGCAGTCGTGCTGCTGTC	PCR detection of SLCMV
SLCMV-PCR-R	TGCTCGCATACTGACCACCA	
SLCMV-A-RTF	ACGCCAGGTCTGAGGCTGTA	Quantification of SLCMV
SLCMV-A-RTR	GTTCAACAGGCCGTGGGACA	
WF-Actin-RTF	TCTTCCAGCCATCCTTCTTG	Quantification of whitefly *actin*
WF-Actin-RTR	CGGTGATTTCCTTCTGCATT	

For agro-inoculation, agrobacteria containing pBINPLUS-1.9A and pBINPLUS-1.9B were cultured separately until the OD600 reached 1.0–1.5. Then bacterial culture was centrifuged at 4000 rpm for 10 min, and the obtained cell pellet was resuspended in resuspension buffer (10 mM MgCl_2_, 10 mM MES and 150 ɥM acetosyringone). Then equal amount (OD) of agrobacteria containing pBINPLUS-1.9A and pBINPLUS-1.9B were mixed. Agro-inoculation was performed with 1 mL syringe when tobacco plants reached 3–4 true leaf stage. Approximately one month later, infection of tobacco plants was examined by inspection of symptoms (Fig. S1) and PCR. Genomic DNA was extracted using Plant Genomic DNA Kit (Tiangen, China) and subsequent detection of viral DNAs was performed with PCR using primers SLCMV-A-PCR-F and SLCMV-A-PCR-R (Table 1).

### 2.3 Virus acquisition and transmission

For virus acquisition, whitefly adults were collected and released onto SLCMV-infected tobacco for a 96 h virus acquisition. When tobacco plants were used as test plants, groups of 10 whiteflies (Asia II 1, MED and MEAM1) were collected and released onto each test plants to feed for 96 h. Three replicates, each containing 10 plants were conducted for each whitefly species. When cassava plants were used as test plants, groups of 30 whiteflies (Asia II 1 only) were collected and released onto each plant to feed for 120 h. Two test plants were used for each of the two cassava varieties used. Leaf-clip cages were used to enclose the whiteflies on the test plants (Ruan et al., 2007). Then whitefly adults were removed and stored in freezer for subsequent determination of infection status using PCR. The test plants were sprayed with imidacloprid at a concentration of 20 mg/L to kill all the eggs. Four weeks post virus transmission, infection of test plants was examined by inspection of symptoms and detection of viral DNAs as mentioned above.

### 2.4 Quantification of virus in whitefly whole body, honeydew and organs

For quantification of SLCMV DNA in whitefly whole body after various virus access periods (AAPs), whitefly adults were collected in groups of 15 and lysed in lysis buffer (50 mM KCl, 10 mM Tris, 0.45% Tween 20, 0.2% gelatin, 0.45% NP40, 60 mg/mL Proteinase K with pH at 8.4) followed by 1.5 h incubation at 65 °C and 10 min at 100 °C to obtain the template for the subsequent virus quantification. Sample preparation of whitefly honeydew after whiteflies have been feeding on infected plants for 48 h and 96 h were conducted as described before (Pan et al., 2018b). For organs, post dissection, four midguts or primary salivary glands were collected as one sample, respectively. Haemolymph from four whiteflies was collected as one sample using the method described before (Pan et al., 2018b). DNA was then extracted using the lysis buffer as mentioned above. Real time PCR was performed using SYBR Premix Ex Taq II (TaKaRa, Japan) and CFX96™ Real-Time PCR Detection System (Bio-Rad, USA) with primers SLCMV-RT-F and SLCMV-RT-R for SLCMV, and primers WF-Actin-F and WF-Actin-R to target whitefly *actin* as a reference gene (Table 1).

### 2.5 PCR detection of SLCMV in whitefly whole body and organs

For PCR detection of SLCMV in whitefly whole body, whiteflies were collected individually after various AAPs. For organs, midguts were dissected and collected individually, and haemolymph from one whitefly was collected as one sample. For primary salivary glands, a pair of them was dissected from the same whitefly and analyzed as one sample. All the samples were then subjected to DNA extraction using lysis buffer as mentioned above and PCR with primers SLCMV-A-PCR-F and SLCMV-A-PCR-R (Table 1).

### 2.6 Immunofluorescence detection of SLCMV in whitefly midguts and primary salivary glands

Immunofluorescence was performed as per the protocol described by Wei et al. (2014) with minor modifications. Midguts and primary salivary glands were first dissected in PBS and fixed for 1 h with 4% paraformaldehyde. Next, the samples were permeabilized with 0.2% Triton X-100 for 30 min, followed by three washes with PBS and a 1 h fixation in 1% BSA dissolved in TBS-Tween 20 (TBST). Organs were incubated overnight with anti-tomato yellow leaf curl virus (TYLCV) monoclonal antibodies (a kind gift from Professor Xueping Zhou, Institute of Biotechnology, Zhejiang University) at a 1:400 dilution at 4 °C. Then the organs were washed and incubated with 549-conjugated secondary antibodies (1:400) (Earthox, China) for 2 h at 37 °C. After washing, organs were covered with DAPI (Abcam, USA) and examined under a Zeiss LSM 780 confocal microscope (ZEISS, Germany).

### 2.7 Statistical analysis

For the quantification of virus in whitefly whole body and organs, all real time data were calculated using 2^-△Ct^ as normalized to whitefly *actin*. For the comparison of transmission efficiency and quantity of virus, normal distribution tests were performed prior to analysis, and then Kruskal-Wallis test was used for analysis of significance. All data were presented as the mean ± standard errors of mean (mean ± SEM). The differences were considered significant when *P* < 0.05. All statistical analyses in the present study were undertaken using SPSS 20.0 Statistics and EXCEL.

## 3 Results

### 3.1 SLCMV transmission efficiencies by three whitefly species

**Fig. 1:**
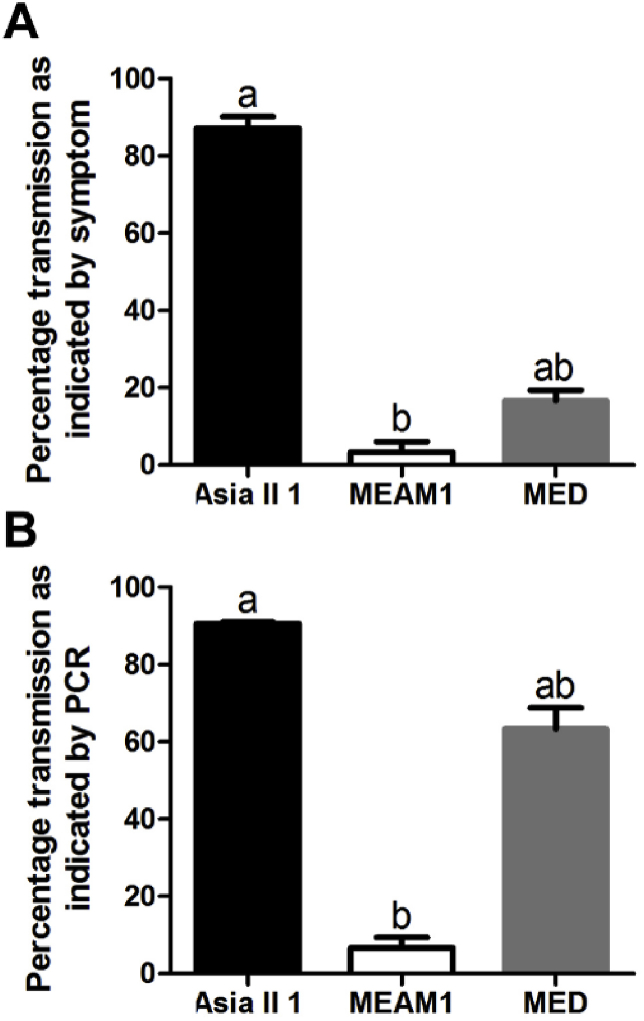
Transmission efficiency of SLCMV to tobacco by three species of the *B. tabaci* complex (Asia II 1, MEAM1 and MED). Whiteflies were allowed a 96 h virus AAP, and then transferred onto tobacco seedlings to transmit the virus for another 96 h. The number of whiteflies per test plant was 10, and for each whitefly species, three replicates were conducted with each consisting of 10 plants. The values represent mean ± SEM of the percentage of PCR positive test plants (A) and percentage of test plants that showed typical symptoms (B) in all plants tested. Different letters above the bars indicate significant differences (Kruskal-Wallis test, *P* < 0.05).

**Fig. 2:**
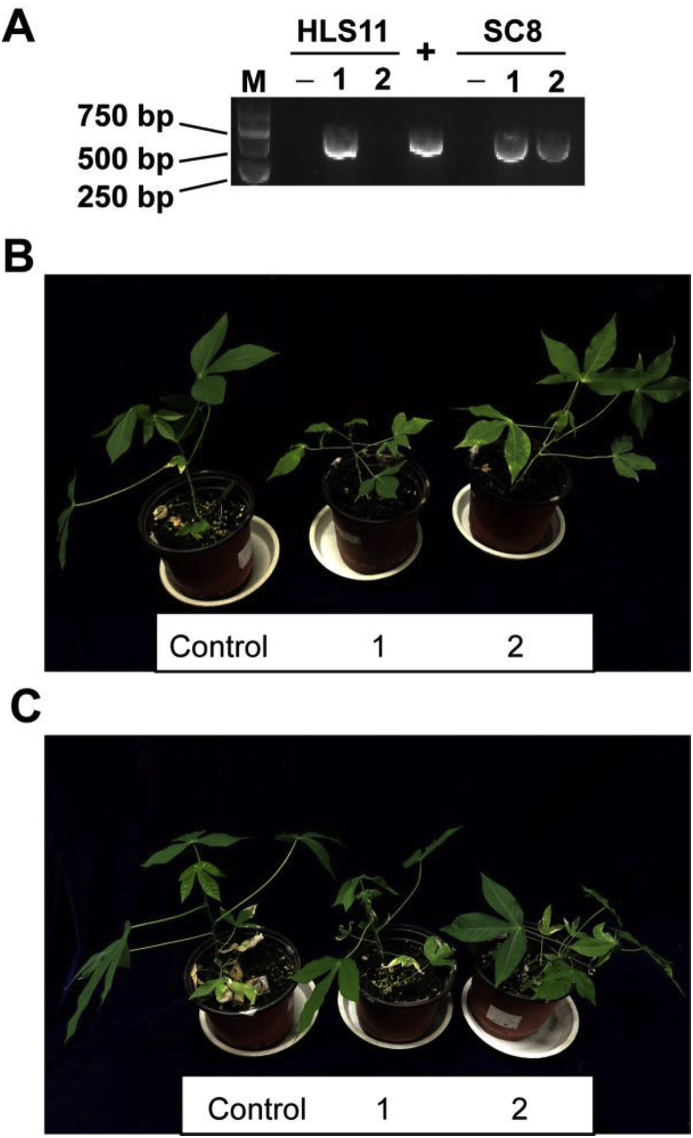
Transmission of SLCMV to cassava (cv. HLS11 and SC8) by Asia II 1 whiteflies. Whiteflies were allowed to acquire SLCMV from SLCMV-infected tobacco plants for 4 days, and then they were collected and released onto cassava seedlings for virus transmission. The number of whiteflies per cassava seedling was 30. Five days later, whiteflies were removed and cassava seedlings were further cultured for another 4 weeks. As for negative control (−), cassava seedlings were kept in a whitefly-free insect-proof cage. Results of PCR detection of SLCMV in cassava plants inoculated by whiteflies were presented in A, and + stands for positive control in PCR analysis. Picture of control and SLCMV-infected HLS11 and SC8 cassava plants were presented in B and C, respectively. As compared to un-infected cassava plants, SLCMV-infected plants exhibited leaf curl and mosaic in young leaves (B and C).

The transmission efficiencies of SLCMV by three species of the *B. tabaci* complex, namely Asia II 1, MEAM1 and MED were compared. The average transmission efficiencies were 87.2% for Asia II 1, 3.3% for MEAM1 and 16.7% for MED as indicated by symptom (Kruskal-Wallis test, χ^2^ = 6.997, df = 2, *P* < 0.05; Fig. 1A). Likewise, the percentages of tobacco plants with detectable SLCMV DNA in all plants tested, differed significantly among the three whitefly species, with the highest transmission (90.5%) by Asia II 1, followed by MED (63.3%) and with only a very low transmission efficiency (6.7%) by MEAM1 (Kruskal-Wallis test, χ^2^ = 7.385, *P* < 0.05; Fig. 1B). Furthermore, to verify the capacity of Asia II 1 whiteflies to transmit SLCMV to cassava plants, we performed virus transmission experiment using two cassava varieties, HLS11 and SC8. As shown in Fig. 2, Asia II 1 whitefly inoculation of cassava plants cv. HLS11 and SC8 resulted in successful transmission of SLCMV, and the transmission rate is 50% and 100% for HLS11 and SC8, respectively.

### 3.2 Acquisition of SLCMV by three whitefly species

**Fig. 3:**
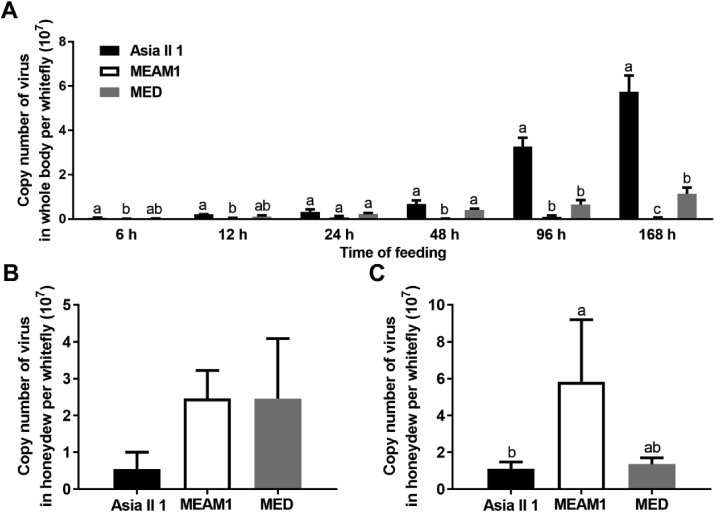
Copy number of SLCMV in whitefly whole body and honeydew. Whiteflies were allowed to feed on SLCMV infected plants, and then at each designated time point, whiteflies were collected and subjected to quantification of SLCMV (A). The honeydew was also collected after whiteflies had been feeding on SLCMV infected plants for 48 h (B) and 96 h (C), and subjected to virus quantification. The number of samples analyzed for each combination of time point and whitefly species is four, and the number of samples analyzed in B or C is eight for each whitefly species. The values represent the mean ± SEM of copy number of virus, and different letters above the bars indicate significant differences (Kruskal-Wallis test, *P* < 0.05).

The copy number of virus in whitefly whole body and honeydew was analyzed by qPCR. While the copy number of virus in the body of Asia II 1 and MED whiteflies seemed to increase with the increase of AAPs, copy number of virus in MEAM1 whiteflies remained at a stable and low level. Furthermore, significant difference of the copy number of SLCMV was found among the three whitefly species except at two points (Kruskal-Wallis tests, χ^2^ = 7.269, 8.346, 9.269 and 9.846 for 6, 48, 96 and 168 h, *P* < 0.05; χ^2^ = 4.750, *P* = 0.093 for 12 h; χ^2^ = 4.500, *P* = 0.105 for 24 h; Fig. 3A). Notably, at all time points checked, the highest copy number of virus was always in Asia II 1, followed by MED, and lowest in MEAM1. Next, the copy number of virus in whitefly honeydew after whiteflies have been feeding on infected plants for 48 and 96 h was analyzed and the results showed that the highest copy number of virus seemed to be present in honeydew from MEAM1, followed by MED and the lowest in Asia II 1 (Kruskal-Wallis tests, χ^2^ = 5.685, *P* = 0.058 for 48 h; χ^2^ = 3.305 for 96 h, *P* = 0.192; Fig. 3B and C).

### 3.3 PCR detection of SLCMV in whitefly whole body and organs

In order to monitor the transport of SLCMV within whiteflies, samples of whitefly whole body and organs were prepared and analyzed after whiteflies were allowed various AAPs (24, 48, 72 and 96 h). As shown in Table 2 for Asia II 1, after 24 h virus acquisition, SLCMV DNA was detected in all whitefly whole body samples and half of midgut samples. With the increase of AAPs, more midgut samples were found to contain detectable amount of SLCMV DNA and viral DNA starts to be detected in haemolymph and primary salivary glands samples after 48 h and 72 h AAPs. Likewise, for MED, SLCMV DNA was detected in all of whitefly whole body samples and some of midgut samples after a 24 h AAP, and viral DNA can be detected in haemolymph after a 72 h AAP. For primary salivary glands, however, no viral DNA was detected in any samples even after a 96 h AAP. For MEAM1, the virus was not found in any samples except in one whitefly whole body sample after a 72 h AAP and one midgut sample after a 96 h AAP.

**Table 2:** PCR detection of SLCMV in whole body, midgut, haemolymph and primary salivary glands of Asia II 1, MEAM1 and MED whiteflies^a^.

Time of feeding	Whitefly species	Whole body	Midgut	Haemolymph	Primary salivary glands
0 h	Asia II 1	0%(0/10)			
	MEAM1	0%(0/10)			
	MED	0%(0/10)			
24 h	Asia II 1	100%(10/10)	50.0%(5/10)	0%(0/10)	0%(0/10)
	MEAM1	0%(0/10)	0%(0/10)	0%(0/10)	0%(0/10)
	MED	80%(8/10)	30.0%(3/10)	0%(0/10)	0%(0/10)
48 h	Asia II 1	100%(10/10)	90.0%(9/10)	30%(3/10)	0%(0/0)
	MEAM1	0%(0/10)	0%(0/10)	0%(0/10)	0%(0/10)
	MED	70%(7/10)	60.0%(6/10)	0%(0/10)	0%(0/10)
72 h	Asia II 1	100%(10/10)	100%(10/10)	40%(4/10)	20%(2/10)
	MEAM1	10%(1/10)	0%(0/10)	0%(0/10)	0%(0/10)
	MED	90%(9/10)	50%(5/10)	10%(1/10)	0%(0/10)
96 h	Asia II 1	100%(10/10)	100%(10/10)	70%(7/10)	60%(6/10)
	MEAM1	0%(0/10)	10%(1/10)	0%(0/10)	0%(0/10)
	MED	90%(9/10)	60%(6/10)	20%(2/10)	0%(0/10)

a Whiteflies were allowed to feed on SLCMV infected plants, and then at designated time points, samples of whitefly whole body, midgut, haemolymph and primary salivary glands were prepared and subjected to PCR. Data are presented as the percentage of PCR positive samples, followed by the number of PCR positive samples and all samples analyzed.

### 3.4 Quantity of SLCMV in whitefly organs

**Fig. 4:**
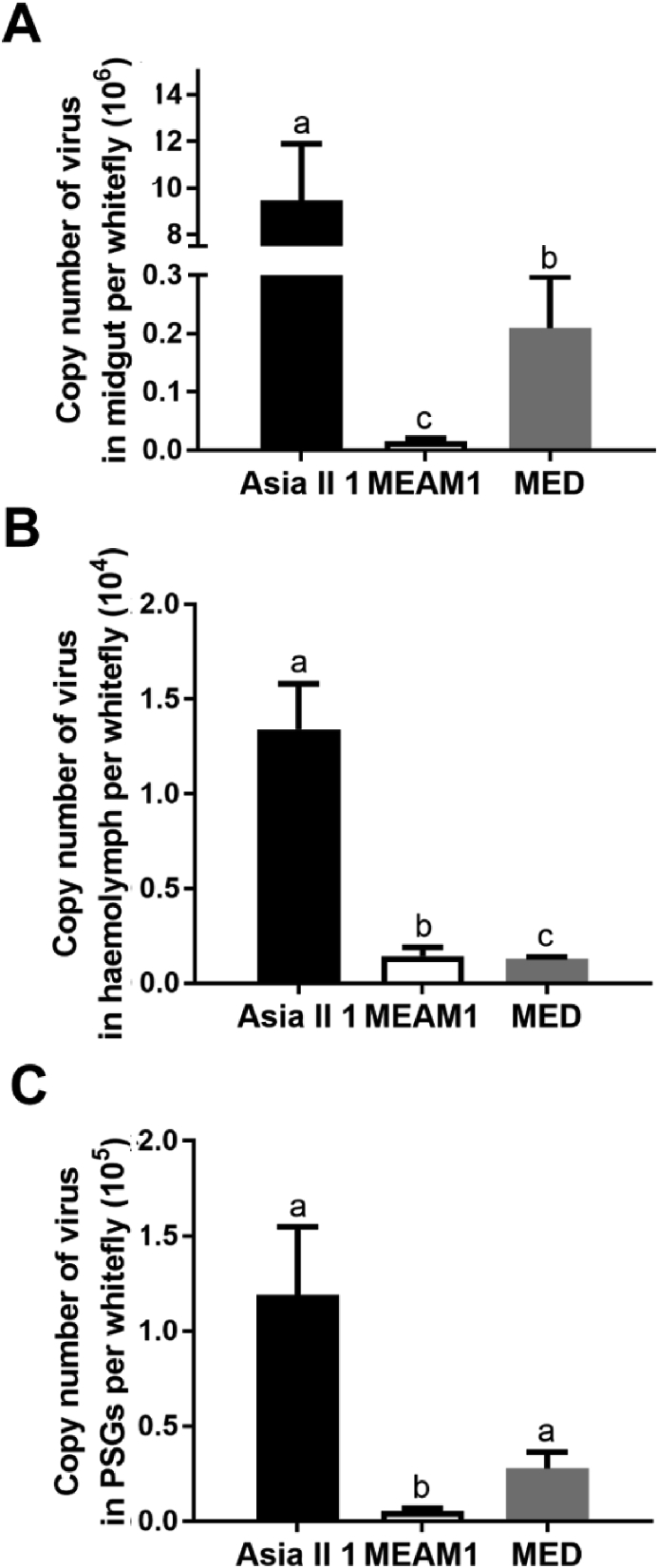
Copy number of SLCMV in whitefly midgut, haemolymph and primary salivary glands (PSGs). After a 96 h AAP, midguts (A), haemolymph (B) and PSGs (C) were collected and subjected to virus quantification. Twelve samples were analyzed for each combination of organ and whitefly species. The values represent the mean ± SEM of copy number of virus. Different letters above the bars indicate significant differences (Kruskal-Wallis test, *P* < 0.05).

After a 96 h AAP, whitefly midguts, haemolymph and primary salivary glands samples were prepared and subjected to SLCMV quantification. In all three organs, the copy number of virus differed significantly among three whitefly species (Kruskal-Wallis test, χ^2^ = 26.495, 24.879, 14.873 for midgut, haemolymph and primary salivary gland, *P* < 0.05 in all cases; Fig. 4). For midgut and PSG, the highest copy number of virus was found in Asia II 1, followed by MED, and the lowest in MEAM1 (Fig. 4A and C). Whereas for haemolymph, the highest copy number of virus was found in Asia II 1, and the copy number of virus in MED and MEAM1 was similar (Fig. 4B).

### 3.5 Immunofluorescence detection of SLCMV signals

**Fig. 5:**
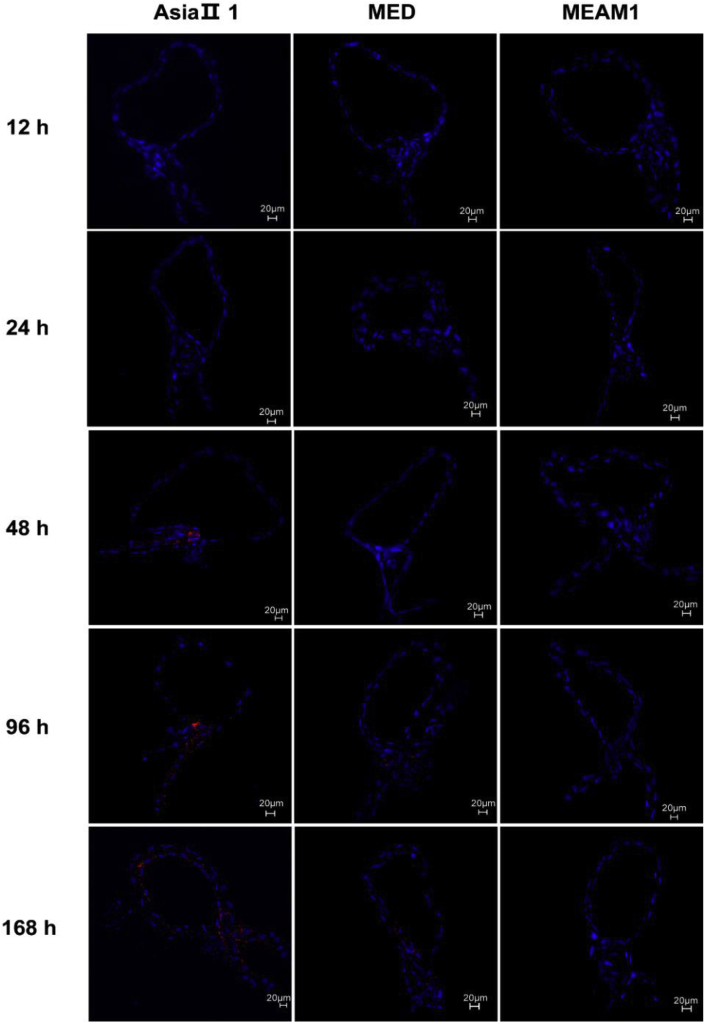
Immunofluorescence detection of SLCMV in whitefly midguts. Whiteflies were allowed to feed on SLCMV infected plants, and then at each designated time point, whitefly midguts were dissected and subjected to immunofluorescence detection of SLCMV. SLCMV was detected using mouse anti-TYLCV antibodies and goat anti-mouse secondary antibodies conjugated to Alexa Fluor 549 (red), and nuclei were stained with DAPI (blue). Images with typical SLCMV signals at each time point are presented.

**Fig. 6:**
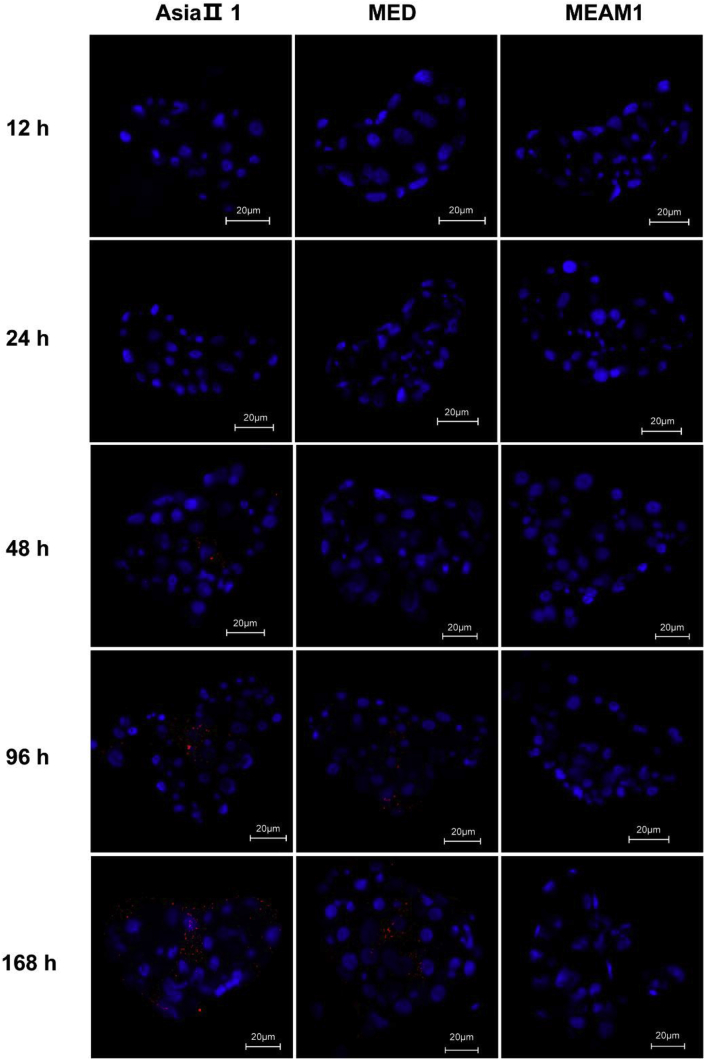
Immunofluorescence detection of SLCMV in whitefly primary salivary glands. Whiteflies were allowed to feed on virus infected plants for various periods of time, and then immunofluorescence was performed. SLCMV (red) and nuclei (blue). Images with typical SLCMV signals at each time point are presented.

Immunofluorescence was used to detect the viral signals in whitefly midguts and primary salivary glands after various AAPs (12, 24, 48, 96 and 168 h). For midguts, while SLCMV signals were detected in the midguts of Asia II 1 and MED whiteflies after 48 and 96 h AAPs, respectively, no viral signal was detected in the midguts of MEAM1 whiteflies even after a 168 h AAP; and in the midguts of Asia II 1 and MED whiteflies, viral signals, mostly found in the filter chamber, became stronger as AAP increased; notably, stronger viral signals were found in midguts from Asia II 1 than those from MEAM1 after whiteflies were given 96 h and 168 h AAPs (Fig. 5). A similar pattern was found when it came to primary salivary glands, with the exception that most viral signals were found in the central secretory region along the ducts of the primary salivary glands (Fig. 6).

## 4 Discussion

In the present study, we compared the transmission efficiency of SLCMV by three whitefly species, and found that while Asia II 1 whiteflies were able to readily transmit the virus, MEAM1 and MED whiteflies poorly transmit SLCMV to test plants to induce symptoms (Fig. 1). Furthermore, the capacity of Asia II 1 whiteflies to transmit SLCMV to cassava plants was verified (Fig. 2). Notably, when tobacco plants were used as test plants, the transmission efficiency of SLCMV by MED whiteflies as indicated by PCR was much higher than that as indicated by symptom (Fig. 1). The possible reasons are: 1) at the time point of examination, the quantity of SLCMV in some MED whiteflies-inoculated plants was not sufficient to induce symptoms but enough to be detected by PCR; 2) MED whiteflies only transferred DNA-A of SLCMV to some test plants. For SLCMV, its transmission by whiteflies has to date only been outlined briefly in two reports, the first of which (Duraisamy et al., 2013) failed to state the species of whitefly successful in transmitting SLCMV. Another study, wherein only a few test plants were used, showed that MEAM1 whiteflies were able to transmit SLCMV from symptomatic cassava plants to tomato and *Arabidopsis thaliana* plants (Wang et al., 2019). Considering the fact that the study by Wang et al. (2019) is a disease note reporting the presence of SLCMV, we believe it is reasonable to state that MEAM1 poorly transmit SLCMV as judged from our data.

The limited capacity of MEAM1 and MED whiteflies to transmit SLCMV suggests that in regions where these invasive whitefly species dominate, e.g., South China, whitefly-borne SLCMV epidemic will hopefully not occur following the recent SLCMV introduction due to the lack of efficient vectors (Hu et al., 2011). Indeed, the same situation was found for CLCuMuV, which was found in South China in 2006 but no major epidemic has been reported, probably due to the limited distribution of its only known efficient whitefly vector, Asia II 1 (Masood et al., 2018; Pan et al., 2018b). Therefore, for the control of SLCMV, in regions where MED and MEAM1 are predominant, thorough implementation of phytosanitary and roguing may be enough to limit the spread of SLCMV. However, in other Asian cassava cultivation areas such as southern Vietnam, multiple indigenous whitefly species including Asia 1, Asia II 1, Asia II 6 have been reported (Götz and Winter 2016). In this regard, research efforts to further examine the transmission of SLCMV by those indigenous whitefly species are important to assist the development of durable control strategies.

In Africa, where CMBs and whitefly species are found to be different from that in Asia, whiteflies of the *B. tabaci* complex seem to play a rather important role in the CMD epidemics (Legg and Ogwal, 1998, Legg et al., 2011, Legg et al., 2014). In a field survey conducted in Uganda in the 1990s, higher populations of whiteflies were reported in epidemic-affected than unaffected areas (Legg et al., 1998). Later, analysis of data from multiple regions in Africa revealed that the spread of severe CMD epidemic generally came after the appearance of ‘super-abundant’ whitefly populations (Legg et al., 2011, 2014). Also, it was established that a distinct whitefly genotype cluster is associated with the epidemic of severe cassava mosaic virus disease in Uganda (Legg et al., 2002). The strong association between the increase of whitefly abundance and presence of severe CMD epidemics suggested that CMD epidemics in Africa might be primarily driven by whiteflies (Legg and Ogwal, 1998, Legg et al., 2002, Legg et al., 2011, Legg et al., 2014). However, in Asia, whiteflies seem to play a more minor role in the epidemics of CMD. As whitefly-borne infection results in symptom appearance in young upper leaves only and cutting-borne infection results in both young and old leaves, field surveys established that whitefly-borne infection was found to account for only 9.0–37.5% and 20.6% of the total incidences observed in India and Vietnam, respectively (Jose et al., 2011; Minato et al., 2019). The reason for the differential role of whitefly in CMD epidemics in Africa and Asia might be the differential transmission of African or Asian CMBs by local whiteflies and/or the abundance of efficient whitefly vectors in regions where CMD occurred. In this regard, a previous study using cassava mosaic geminiviruses and whitefly populations collected from India and Africa established that cassava mosaic geminiviruses from either location are transmitted efficiently only by whitefly populations from their geographical origin (Maruthi et al., 2002). Therefore, it is tempting to speculate that the lack of efficient CMB vector populations might account for the limited whitefly-borne infection in Asia.

For the role of whitefly vectors in CMD epidemics, while it has been well established in the African context, much more remains to be explored in Asia (Legg et al., 2002, 2011, 2014). In Cambodia and Vietnam, the outbreaks of CMD caused by SLCMV were found to be associated Asia II 1 whiteflies, the only known efficient vectors for SLCMV as we revealed in the present study (Wang et al., 2016; Uke et al., 2018). These findings provide valuable insight into the role of whitefly in Asian CMD. However, more studies, which should include detailed comparison of whitefly distribution and abundance in Africa and Asia, and comparative transmission of more different whitefly species-CMB combinations, are necessary to further illustrate the reasons for the differential role of whitefly in the outbreak of CMDs in the two continents.

Further, in order to explore the mechanisms underpinning the differential transmission of SLCMV by different whitefly species, we monitored virus acquisition by and virus transport inside whiteflies. Our findings revealed that the transmission efficiencies of SLCMV by different whitefly species correlated positively with quantity of virus in whitefly whole body, but negatively with that in honeydew. It was also noted that the variation of transmission efficiency was associated with the differing virus transport inside whitefly, particularly across the whitefly midgut. Interestingly, the pattern of differential transmission of SLCMV and underlying mechanisms are similar to that of CLCuMuV and tobacco curly shoot virus (TbCSV) when only Asia II 1 and MEAM1 are considered, suggesting something in common in those three viruses, probably in their coat proteins considering the function of coat proteins (Briddon et al., 1990; HöFer et al., 1997; Czosnek et al., 2017; Harrison et al., 2002; Pan et al., 2018, Pan et al., 2018b). For begomoviruses, once they are acquired by insect vectors during feeding, they move long the food canal and then translocate from the gut lumen into the haemolymph and finally into the salivary glands, from where they are introduced back into the plant host during insect feeding (Czosnek et al., 2017; Hogenhout et al., 2008). Therefore, the information provided here, along with those in previous reports, offers a unique opportunity to further explore the nature of virus transport within whitefly and factors involved, e.g., the motifs of coat protein involved in whitefly-begomovirus interaction, thereby advancing our understanding of whitefly transmission of begomoviruses.

Taken together, here we show that indigenous Asia II 1 whiteflies were able to readily transmit SLCMV and invasive MEAM1 and MED whiteflies can only transmit this virus with very low efficiency. Further analysis revealed that the differential transmission might be due to the differential capacity of SLCMV to be retained by different whiteflies and to transport across the midgut of different species of whiteflies. To the best of our knowledge, this study is the first to explore the detailed whitefly transmission profile of an Asian CMBs. Our findings identified Asia II 1 whiteflies, but not MEAM1 or MED whiteflies as efficient vectors for SLCMV, which will help to evaluate the potential threat of SLCMV to cassava production in many regions and to facilitate the prediction of virus epidemics.

## Author contributions section

Yao Chi: Conceptualization, Methodology, Formal analysis, Investigation, Data Curation, Writing - Original Draft, Writing - Review & Editing.

Li-Long Pan: Conceptualization, Methodology, Formal analysis, Data Curation, Writing - Original Draft, Writing - Review & Editing.

Sophie Bouvaine: Conceptualization, Validation, Investigation, Resources, Writing - Review & Editing, Supervision, Project administration, Funding acquisition.

Yun-Yun Fan: Investigation.

Yin-Quan Liu: Resources, Supervision, Project administration, Funding acquisition.

Shu-Sheng Liu: Conceptualization, Resources, Supervision, Project administration, Funding acquisition.

Susan Seal: Conceptualization, Validation, Investigation, Resources, Writing - Review & Editing, Supervision, Project administration, Funding acquisition.

Xiao-Wei Wang: Conceptualization, Resources, Writing - Review & Editing, Supervision, Project administration, Funding acquisition.
