# Supplementary material for: Differential transmission of Sri Lankan cassava mosaic virus by three cryptic species of the whitefly Bemisia tabaci complex
Source: Virology. 2020 Jan 15;540:141–9. doi: 10.1016/j.virol.2019.11.013 (PMC6971692; doi:10.1016/j.virol.2019.11.013)
Supplement: Multimedia component 2 [file mmc2.docx]

>SLCMV DNA A

>ACCGGATGGCCGCGCCCCCCGCTTTGTGGTGGACCCCCCCCCCACGTGGAGATGTCCCCCACTCAGAACGCTCCCTCAAAGCCTGTATAGTTGTGGTCCCTCTTTAAGTACTTGCTCAGCAAGTTGTAATCTGCACAATGTGGGACCCTTTGGTAAACGAGTTCCCTGAATCAGTTCACGGTTTCCGGTGTATGCTTGCCGTGAAATATCTTCAGCTAGTTGAAGGTACTTATTCCCCCGATACACTCGGTTACGATTTAATCAGAGATTTGATCTCTGTCATCAGGGCCAAAAATTATGTCGAAGCGACCAGCAGATATAATCATTTCAACTCCCGCCTCGAAGGTTCGTCGCCGTCTGAACTTCGACAGCCCATACAGCAGTCGTGCTGCTGTCCCCACTGTCCGCGTCACAAAAAGACAAGCCTGGACAAACAGGCCCATGAATCGGAAGCCCAGGTGGTACAGGATGTTCAAAAGCCCAGATGTTCCTAGGGGATGTGAAGGCCCATGTAAGGTTCAATCGTTTGAGTCCAGACACGATGTGGTCCATATAGGTAAGGTCATGTGCATCTCTGATGTCACTCGTGGAGTTGGGCTTACTCATCGCGTGGGTAAGAGGTTTTGCGTTAAGTCCGTTTATATCCTGGGTAAGATATGGATGGATGAAAATATTAAGACCAAGAATCATACGAATAGTGTGATGTTCTTCCTTGTAAGGGATCGTAGGCCTGTTGATAAGCCCCAGGATTTTGGTGAAGTGTTTAATATGTTCGATAATGAACCTAGTACAGCTACGGTGAAGAACATGCATCGTGATCGTTATCAAGTCCTCAGGAAGTGGAGTGCCACTGTCACTGGTGGTCAGTATGCGAGCAAGGAACAGGCTTTAGTTAGGCGTTTTTTTAGAGTTAATAATTATGTTGTGTATAACCAGCAAGAGGCTGGCAAGTATGAAAATCATACCGAGAATGCATTGATGCTGTACATGGCGTGTACTCATGCCTCTAACCCTGTATACGCTACGCTGAAGATTAGAATCTACTTCTACGATTCGGTCAGCAATTAATAAACATTAAATTTTATTAAATTAGACTGCTCAATACTGTCAGTCCCAGCGATTACATTATATAATACATGTTCTACTGCGTTTACAACCGTATTAATACATAGAATCCCTAATCTATCGAGGTATTTTAATACTTGGGTCTTAAATACCCTCAAGAAACGCCAGGTCTGAGGCTGTAAGGTCGTCCAGACCTTGAAATCCATCCAGCATTGATGTAGTCCCAACGCTTTCCTCAGGTTGTGGTTGAAGCGTATCTGGATGGTTATTATGTCCCACGGCCTGTTGAACGGCCGGCTGTCGTGCTGGATGATCCTGAAATAGAGGGGATTTGGAACCTCCCAGATATATACGCCATTCATCGCCTGAGCTGCAGTGATGAGTTCCCCTGTGCGTGAATCCATGGTTGTGGCAGTTGATGTGCACGTAGTATGAGCAGCCGCAATTGAGGTCTACTCTCCGTCGCCGAATGGCCTTACGCTTAGCTGCCCTGTGTTGGACCTTGATTGGAACCTGAGTAGAGCGGCTCGCTGAGGGAGATGAAGGTCGCATTCTTCAGAGCCCAAGCCTTCAATGCGCTATTCTTCGCCTCGTCAAGGAATTCTTTATAGCTGGAATTGGGCCCAGGATTGCAGAGGAAGATAGTGGGAATTCCCCCTTTAATTTGAACTGGCTTCCCGTACTTGGTGTTTGACTGCCAGTCTCTTTGGGCCCCCATGAATTCCTTAAAGTGCTTTAGATAGTGCGGATCTACGTCATCAATTACGTTGTACCATGTATCATTGCTGTAAACCCTAGGACTCAAGTCCAGATGTCCACACAGATAATTGTGTGGACCTAATGACCTAGCCCACATTGTTTTGCCTGTACGACTATCGCCCTCGATGACTATACTATTAGGTCTCAAAGGCCTCGCAGAGGCACCCATGACGTTCTCGGACACCCACTCCTCAAGTTCATCTGGAACTTGGTCAAATGAAGAGGCTGAGAAGGGAGACACATATACCTCGGGAGGAGGTGTAAAAATCCTATCTAAATTAGCATTTAGATTATGAAATTGTAAAACATAATCCTTGGGTGCTAACTCCTTAATGACTCTAAGAGCCTCTGGCTTACTGCCTGTGTTAAGTGCTGCGGCGTAAGCGTCGTTTGCTGACTGCTGTCCCCCTCTTGTAGATCGTCCATCGATCTGAAACTCACCCCAGTCGATGGTGTCTCCATCCTTGTCGATGTAGGACTTGACGTCTGAGCTGGACTTAGCGCTCTGTATGTTGGGGTGGAAACTGGTGCTACTGCTTGGGTGTACACAATCGAATTGCCGATTGTTTGTGATTGTGAGCTTCCCTTCGAACTGAAGCAGAGCATGGAGGTGAGGTTCCCCATTCTGATGGAGTTCTCTGCAAATTTTAATAAATTTGATGTTTGTCGGAAGACTCAAGCTTCGGAAGAACTCGAGTAAGTGTTCTTTGGTGAGAGAACACTTAGGGTATGTGAGGAATATATTTTTGGACTGAATTCTGAATCGTGGGTTTCTCATCTTTGACTCGGTCAATTGGAGACACTCCTGAGCAAGTCTCTAGTGAATTGGAGACAATATATATGTGTCTCCAAATGGCATTCTTGTAATTCTCAAAAGTTACAGTCAAATTTCAAATTTGAATTTGAGATCCAAAAGCGGCCATCCGTATAATATT

>SLCMV DNA B

>ACCGGATGGCCGCGCCCCCCTCTTTGTGGTGGCCCCCCCCACGTGGGGATGTCCCCCTCTCACAACGCTCACTAGAAGGTTCAACATGTTGGTGGCCCCACGATGTTGTTTATAACGTCTATAACGTTTGAGACTCGAAGCTTGTGATGCCACGTATGCGTTATTTGTACTTCGTCTCGAAGTTATGAGTGAGGGCCCTATATGTGGATATCTAACCGCATATTGTGTGAATGGATTAAACGTGGCAAGATCATGCCGTTTATTGGGAACTATATTATTATGCTGTCGTCTATATATATAGGTATGTCGTGTTTCGGAGATCTGCACATGGTGGTGGCATGATGAGAAGAGGTGCCTATACCCCCCGTTCTACTCCATTCTCTCGTGACCGGAGATCGTATAATGCCGGTAAGGGTAGATCATTTCGTTCTTACCGTCGTCGTGGACCTGTTCGTCCATTAGTTCGTCGGAACCTGTTTGGTGATGACCATGCACGTGCATTTACGTATAAGACCGTATCGGAGGATCAATTTGGACCGGATTTTACCATACATAATAATAATTATAAGTCATCGTATATATCTATGCCTGCCAAAACACGTGCCCTTAGCGATAACAGGGTAGGTGATTATATCAAACTTGTAAATATATCATTTACAGGTACAGTGTGTATTAAAAACAGCCAGATGGAATCTGACGGAAGCCCAATGTTGGGCCTGCATGGGCTGTTTACTTGTGTATTGGTCCGGGATAAGACCCCTCGTATATATTCTGCCACTGAGCCTTTGATACCTTTCCCACAGTTGTTTGGGTCCATAAACGCGAGCTATGCGGATTTGTCTATACAAGACCCATATAAGGATCGGTTCACAGTTATCCGTCAGGTGTCTTACCCAGTTAATACGGAGAAGGGTGATCATATGTGTCGTTTCAAAGGCACTCGACGTTTTGTTGGTAGATACCCTATCTGGACTAGTTTTAAAGATGATGGTGGCATTGGAGATTCATCGGGATTATATAGTAATACGTATAAAAATGCCATACTTGTATATTATGTATGGCTCAGCGACGTATCGTCACAATTGGAAATGTATTGTAAATATGTAACTCGATATATTGGTTAATAAAAATGTTATACATTTTTGGATACATGGCTCTGCATACTCGTATTTAAACACATATGTACTGTCTTGCTGATGATGTCGTTTAAGTCCTCTCGTGTGAAGTTGTCCGATCCTATTTGTGATACTGACTTTCCGGGGTCCAATGCGTCTGGGTTGAGCCGGTTTAGTTGGCTGTAAGGCTTTTCAATTGATGGCCCAGCCTCGCTCTTAACGGCCCATGGCTCGTGAAGCCCAACCTCGCTTCTAACGGCCCATGATTCATTAGGGCCTATTGAACATGGAGTATATCTCATGGATCTGGATCCTATTAGACTTGGGCCTTGGACCAGTTTCCTCTGCTGGGGTTTCCGTCCCACTGACCAGAAATCAATGTCCCTCTCAGTGAAGGCCTTGCTCAGTATTTCGATTTTGGGAGATCGGAACTGTATGTCATTAGACTGCTTTGCAGTTGACAGCTTGAGTTTTCCCTGTATACGACAGAAGTGGACCCCGTTGATGACGTTTGTGTCTACGACCCTGTACATAACCCTCCATGGGTTTATGTCCTTCATGGAGAAGAACGATGAAGAATAGTAGTGAAGGTTGCAGTTGCATTGGACGGGAATAGTAAATTCTGCCTGTTTTGAGTCTCCGTCGTGCAGTCTTTGGTCGTGAATTTCAATGATGACATGCCCTGTGGCGTTAATGGGTACCTGGTTTCTATACTCTAGGACGATGTGGTCTATTTTGCAGCAGTGACCCTTGAGGAGCGATATTTTGTTATCCAGAAGAGATGGAAAACTCAACTTGACGTCGGTTGAGTCATTGGTTAACTCATATTCAACTCTTTCGGAACGAAGATACGCTGCATTGCTACTATTATTCTCCATTGGCCCCGCAGGGGAAATGCTTAAAAATTAAGCCCAGTGCAGAAGAAATAATTTAATGTGCATAAAGCAAAGCCCAGAGCTCAATAAATAGCTAAAGGCATATATATTATATTATTGATGAAAGGTTACAAGAGCATCCACGTGGCATTGGATTAAATACATGAAAAGCTATACAATATATTTAAAAAAAGTTGTGACGACGTCATCTTCAACGAATGGAACCGGAAACCCTAATGCATCTCTGATGACTCTGGTTAAGCCCTCTAGTAGTATGTCACATGTTTCCGGTGTCATGTGGTAGTAATTTTTGAGTTGGTAACCCCAATCTTCCAATTTGAATGCCATATGTATGGCTCTATCTATTTCTGGTAACAGATCCATATCTTGTTCTTCGAAGTGGAGGTAATAGGAATTTGAACCAGTCGCGATGTAAGGTTGATGTCTGCTAGTAGTTTTGAATTCGACCCAGATCGAATTCTTCATAATGCGACTGGATAGTCGCACACATTTCTTGCTGTGTTGCAAGAAGGAATAGTTAGTTTGGGAAGACATAGGAAGAATGAGAATGATGTTGGGTTTAAGTGGGATTTCTCTCTCCTCAATTGGAGACACCTCTGAGCAAGTCTCTAGTGAATTGGAGACAATATATATGTGTCTCCAAATGGCATTCTTGTAATTCTCAAAAGTTACAGTCAAATTTCAAATTTGAATTTGAGATCCAAAAGCGGCCATCCGTATAATATT
